# Broadband frequency translation through time refraction in an epsilon-near-zero material

**DOI:** 10.1038/s41467-020-15682-2

**Published:** 2020-05-01

**Authors:** Yiyu Zhou, M. Zahirul Alam, Mohammad Karimi, Jeremy Upham, Orad Reshef, Cong Liu, Alan E. Willner, Robert W. Boyd

**Affiliations:** 1 The Institute of Optics, University of Rochester, Rochester, NY 14627 USA; 2 Department of Physics, University of Ottawa, Ottawa, ON K1N 6N5 Canada; 3 Department of Electrical Engineering, University of Southern California, Los Angeles, CA 90089 USA

**Keywords:** Metamaterials, Nonlinear optics

## Abstract

Space-time duality in paraxial optical wave propagation implies the existence of intriguing effects when light interacts with a material exhibiting two refractive indexes separated by a boundary in time. The direct consequence of such time-refraction effect is a change in the frequency of light while leaving the wavevector unchanged. Here, we experimentally show that the effect of time refraction is significantly enhanced in an epsilon-near-zero (ENZ) medium as a consequence of the optically induced unity-order refractive index change in a sub-picosecond time scale. Specifically, we demonstrate broadband and controllable shift (up to 14.9 THz) in the frequency of a light beam using a time-varying subwavelength-thick indium tin oxide (ITO) film in its ENZ spectral range. Our findings hint at the possibility of designing (3 + 1)D metamaterials by incorporating time-varying bulk ENZ materials, and they present a unique playground to investigate various novel effects in the time domain.

## Abstract (web summary)

Here, the authors present an experimental demonstration of adiabatic frequency conversion using the concept of time boundary by exploiting the properties of an ITO film operating near its epsilon-near-zero frequency. They demonstrate a large and controllable shift up to 14.9 THz.

## Introduction

**Fig. 1:**
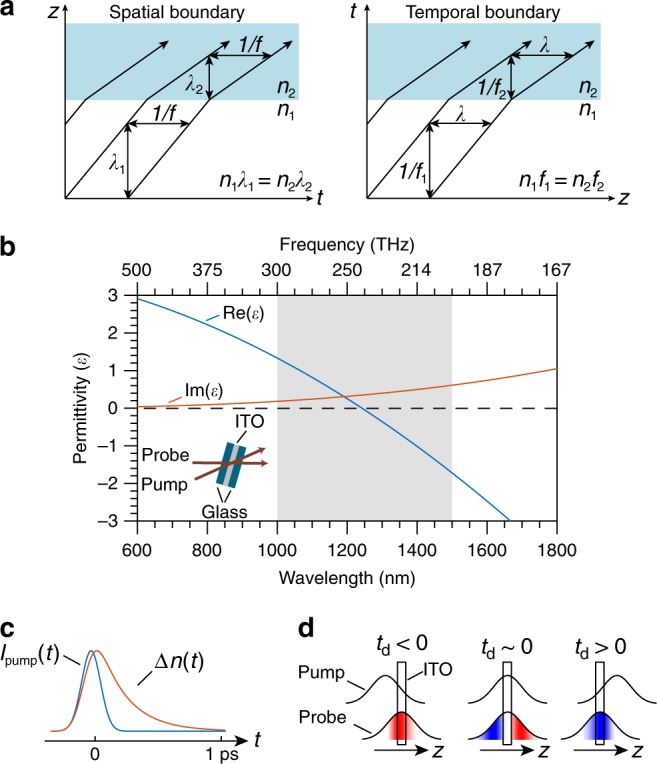
Concept of time refraction. **a** A spatial boundary defined by a refractive index change from *n*_1_ to *n*_2_ leads to a change in the wavevector of a light beam as it passes through the boundary and is described by *n*_1_*λ*_1_ = *n*_2_*λ*_2_ (left panel). A refractive index boundary defined in time leads to time-refraction effect of a light beam as it passes through the boundary and is described by *n*_1_*f*_1_ = *n*_2_*f*_2_ (right panel). Here *f* is the frequency of light waves in the medium. **b** The permittivity of an ITO film used in the experiment. The inset shows the simplified experimental setup and the shaded region shows the spectral range of interest in this work. **c** Simplified illustration of the temporal index change Δ*n*(*t*) of ITO excited by a pump pulse. **d** The frequency of the probe redshifts (blueshifts) if the pump beam lags (leads) the probe. At near-zero delay both redshift and blueshift can occur.

Maxwell’s equations describe how an electromagnetic wave is modified by a material. The spatial boundary condition associated with Maxwell’s equations can be used to derive the well-known Fresnel equations and Snell’s law. A spatial variation in refractive index leads to reflection and refraction of a light beam incident on the boundary. As a consequence, the wavevector of the transmitted light changes, whereas the frequency is conserved. The spatial boundary can be abrupt (nonadiabatic) in refractive index variation such as at a glass-air interface. Or, the boundary can be smoothly varying, i.e., adiabatic in space, such as in a gradient-index lens. In both cases, the refracted beam of light must have a different *k*-vector (Fig. 1a), where |*k*| = 2*πn*/*λ*, *n* is the refractive index of the medium, and *λ* is vacuum wavelength of light. As the equations describing the paraxial wave propagation are unchanged upon the interchange of time and a spatial coordinate, one can define a boundary of refractive index in the time coordinate in a dual fashion to that in the spatial coordinates^1–5^. This effect is known as time refraction.

The concept of time refraction is presented in Fig. 1a. Let us assume that an optical pulse of frequency *f*_1_ is traveling in a dispersionless medium with a refractive index of *n*_1_. At *t* = *t*_1_ the refractive index changes from *n*_1_ to *n*_2_. As a consequence of the broken time translation symmetry, the frequency of light has to change because of the change in the refractive index while leaving the wavevector unchanged^6^. The change in frequency, according to the dispersion relation *c*/*f* = *nλ*^7^, can be expressed as *n*_1_*f*_1_ = *n*_2_*f*_2_ = (*n*_1_ + Δ*n*)(*f*_1_ + Δ*f*), where Δ*f* = *f*_2_−*f*_1_ is the change in the frequency of light after it encounters the temporal boundary; Δ*n* = *n*_2_−*n*_1_ is the change in the refractive index; and *c* is the speed of light in vacuum. Consequently, we can express the change in frequency as Δ*f* = −Δ*n*·*f*_1_/(*n*_1_ + Δ*n*). Thus, the frequency shift may be red (blue) if the change in index Δ*n* is positive (negative). This effect is strongest when Δ*n*/(*n*_1_ + Δ*n*) is large. In a regular dielectric medium such as silicon^8^, Δ*n*/(*n*_1_ + Δ*n*) can only be on the order of 10^−3^. In contrast, in a highly nonlinear low-index medium, Δ*n*/(*n*_1_ + Δ*n*) can approach unity due to the near-zero linear refractive index *n*_1_ and the large nonlinear index change Δ*n*^9–11^. Thus, a highly nonlinear low-index medium is a natural platform with which to generate a large frequency translation using time refraction. In addition to frequency conversion, a time-varying medium with a large index change can also be used to investigate many novel effects in the time domain such as all-optical nonreciprocity^12,13^, negative refraction^14^, photonic topological insulators^15^, photonic time crystals^16^, achromatic optical switches^17^, and the dynamic Casimir effect^18^.

A number of effects have been used to experimentally implement a time-varying medium, such as free-carrier dispersion^19–24^, Kerr nonlinearity^25–28^, laser-induced plasma^29–32^, and optomechanical interaction^33^. The magnitude of frequency conversion in a time-varying medium fundamentally depends on the available index change. This is in contrast to other nonlinear optical effects such as four-wave mixing^8^, where the constraints of weak nonlinearity can be almost entirely overcome through use of a very long interaction length^34^. Resonant structures such as micro-ring resonators and slow-light photonic crystals, tend to exhibit enhanced sensitivity to the change in the material’s refractive index. Such resonant structures can be used to somewhat sidestep the restrictions imposed by the intrinsically low nonlinearity of materials to obtain appreciable adiabatic frequency conversion (AFC)^19–22,35–39^. Using these techniques, adiabatic frequency conversions up to 280 GHz (or ~0.145% of the carrier frequency) have been previously demonstrated^20^. Nevertheless, all prior demonstrations of AFC have exhibited the following limitations: narrow operational bandwidth^20,34,38,39^; relatively long interaction length^19,21–25,33,36,37,40^; limited tunability with respect to the magnitude and sign of shift^19–24,40^; the requirement of inhomogeneous structure limiting wide adoptions into various platforms^19–24,33,35–39^; and the possible requirement of out-of-plane above-bandgap excitation pulses^20^.

Here, we show that we can simultaneously overcome all of the above-mentioned shortcomings by using a homogeneous and isotropic epsilon-near-zero (ENZ) medium of subwavelength thickness. Using a series of pump-probe measurements, we demonstrate optically controlled total frequency translations of a near-infrared beam of up to 14.9 THz (redshift of 11.1 THz and blueshift of 3.8 THz)—that is, over 6% of the bandwidth of the carrier frequency—using a 620-nm-thick ITO film. The effect of frequency translation is broadband in nature, i.e., the central wavelength of the degenerate input pump and probe pulses can be tuned over a 500 nm range. We also find that the effect is maximum near the zero-permittivity wavelength of ITO.

## Results

### Nonlinear optical response of the ENZ material

An ENZ material is defined as a medium that has a near-zero linear permittivity, and consequently low linear refractive index. The near-zero permittivity in such a medium leads to highly nonintuitive linear effects^41–43^ and strong nonlinear light-matter interactions^9,44–47^. In order to implement a temporal boundary with a large index change, we make use of the large and ultrafast optically induced change in refractive index of a 620 nm thick ITO film in its near-zero-permittivity spectral range. ITO is a degenerately doped semiconductor and near its zero-permittivity wavelength (1240 nm), the linear permittivity of the ITO sample can be well described by the Drude model (Fig. 1b). The temporal nonlinear optical response of ITO can be described by the two-temperature model when excited by an optical pulse with a central wavelength close to the ENZ region^9,44^. The optical excitation of ITO near the ENZ region leads to a strong modification of the Fermi-Dirac distribution of the conduction band electrons. The highly nonequilibrium distribution of electrons, within the formalism of the Drude model, leads to an effective redshift of the plasma frequency owing to the momentum-dependent effective mass of the electrons. According to the two-temperature model, the rise time of the change in the refractive index is limited by the thermalization time of the conduction band electrons owing to electron-electron scattering. The rise time also depends on the energy deposition rate in the ITO film and thus has a strong dependence on the temporal envelope of the pump pulse. Once the pump pulse peak leaves the ITO film, the index returns to the initial value within a sub-picosecond time scale through electron–phonon coupling (Fig. 1c). Owing to the time-dependent nature of the index change induced by the intensity of the pump pulse, the frequency of probe pulse can be redshifted or blueshifted depending on the pump-probe delay time (see Fig. 1d).

### Measurements at the near-zero-permittivity wavelength

**Fig. 2:**
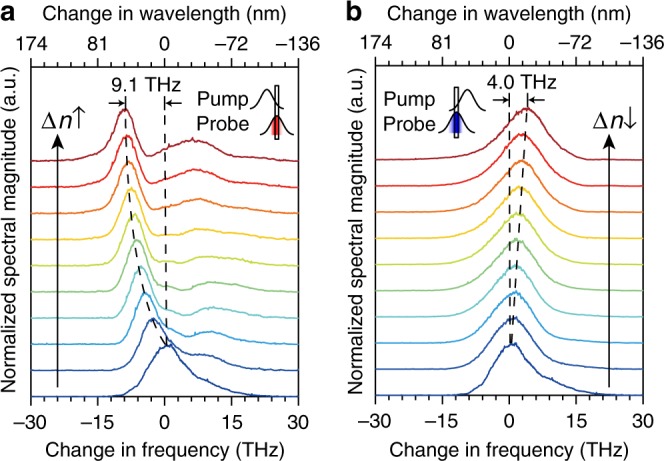
Pump-induced frequency translation at a fixed delay time. **a**–**b** The frequency of a 1235 nm probe beam redshifts at the delay time *t*_d_ = −60 fs in **a** and blueshifts at the delay time *t*_d_ = 60 fs in **b**. The insets show the relative position of the pump and the probe. The top-most (bottom-most) spectra in both panels correspond to the largest (zero) pump intensity and, consequently, the largest (zero) change in the refractive index.

In order to measure the magnitude of the frequency translation using ITO, we performed a set of degenerate pump-probe experiments with ~120 fs pulses and recorded the spectra of the probe beam as a function of the delay between the pump and the probe for varying pump intensities. The ITO film has two 1.1-mm-thick glass slabs on both sides. Both pump and probe beams are *p*-polarized, and the intensity of the probe beam is kept low to avoid nonlinear effects (See Methods and Supplementary Note 1 for more details). The results for *λ*_0_ = *λ*_pump_ = *λ*_probe_ = 1235 nm at the pump-probe delay time of ±60 fs is shown in Fig. 2. The pump induces a nonlinear change in the refractive index of ITO with a rate that depends on the pump intensity, the temporal envelope of the pump, and the intrinsic nonlinear dynamics of the ITO. When the pump pulse is delayed with respect to the probe, i.e., pump-probe delay time *t*_d_ < 0, the probe experiences a rising refractive index and thus its spectrum redshifts (Fig. 2a). If the probe reaches the ITO after the peak of the pump pulse is passed (*t*_d_ > 0), it experiences a falling refractive index change and the spectrum of the probe blueshifts (Fig. 2b). We also note that for *t*_d_ ≈ 0 both blueshift and redshift can occur (Fig. 1d). As the thickness of the ITO film is only 620 nm, 120 fs pump, and the probe pulses never reside entirely within the ITO thin film (Fig. 1d). Thus, the magnitude of the frequency shift of the probe pulse becomes dependent on the index change rate Δ*n*/Δ*t* it experiences while transiting through the ITO film. We extract the effective values of the index change rate based on the experimental data through numerical simulations (see Supplementary Note 2). In numerical simulation we use the slowly varying envelope approximation and, as a result, the predictions of our model are only dependent on the envelope-averaged dynamics of the ITO.

**Fig. 3:**
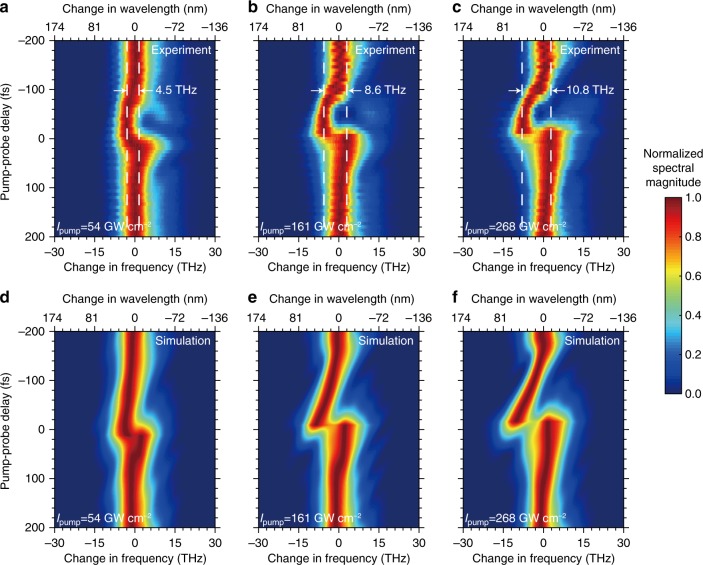
Experimental and simulated probe spectra at *λ*_0_ = 1235 nm. **a**–**c** Experimental probe spectra as a function of the pump-probe delay time for varying pump intensities. The spectral magnitude for each pump-probe delay is normalized individually. **d**–**f** The corresponding numerically simulated probe spectra modeled by the nonlinear Schrödinger equation. The spectra of the probe show a strong dependence on the pump intensity and pump-probe delay time. For a pump intensity of 268 GW cm^−2^, the total frequency translation at this wavelength is 10.8 THz.

We find that both the pump intensity and the value of the pump-probe delay time modify the spectra of the transmitted probe. We present the results for *λ*_0_ = 1235 nm for three pump intensities in Fig. 3a–c. In general, the time refraction leads to the modification of amplitude, bandwidth, temporal width, and the carrier frequency of the probe pulse (see Supplementary Note 3). In order to focus on the spectral shift, the magnitude of spectrum for each pump-probe delay value is individually normalized in Fig. 3. We find that when the absolute value of the pump-probe delay time |*t*_d_| is increased, the magnitude of the frequency translation for the probe decreases. Furthermore, when pump-probe delays are small, the leading portion of the probe pulse experiences an increase in refractive index (thus redshifts), whereas the trailing portion experiences a decrease of refractive index (thus blueshifts). This is evident in Fig. 3a–c by the presence of two peaks at *t*_d_  ≈ 0. For a fixed pump-probe delay time an increase in pump intensity leads to larger change in index and, as a result, a larger shift in the central frequency of the probe pulse. Furthermore, we find that the fall time of the index change is slower than the rise time of the index change. The fall time of the index change is longer because it—within the formalism of the two-temperature model—is dictated by the intrinsic electron–phonon coupling rate, the maximum temperature of the conduction band electrons, and the thermodynamical properties of the lattice. As a result, the rate of decrease in index after the pump leaves the ITO film is smaller compared with that of the rising edge, and therefore the magnitude of the achievable redshift for a constant pump intensity is larger than the achievable blueshift. At a sufficiently high pump intensity, we observe an appearance of a large blueshifted spectral peak when the pump is at 1235 nm owing to higher-order nonlinear optical effects. At a peak pump intensity of 483 GW cm^−2^ the blueshift can be as large as 10.6 THz (~52 nm in wavelength), and the total maximum frequency translation can be larger than 20 THz (see Supplementary Note 4). This value corresponds to a fractional frequency shift (Δ*f*/*f*_0_) of ~9%.

We model the time-refraction effect in ITO using the nonlinear Schrödinger equation, and the split-step Fourier method is used to numerically solve the Schrödinger equation^48^. We use an iterative algorithm to calculate the approximate shape of the time-varying nonlinear phase variations induced by the index change to fit the experimentally measured spectra (see Supplementary Note 2). The simulation results are shown in Fig. 3d–f. Our numerical model is in excellent agreement with the experimental data, confirming that the origin of the shift is owing to the rapid change of index experienced by the probe pulse while transiting through the ITO sample.

### Measurements over a broad spectral range

**Fig. 4:**
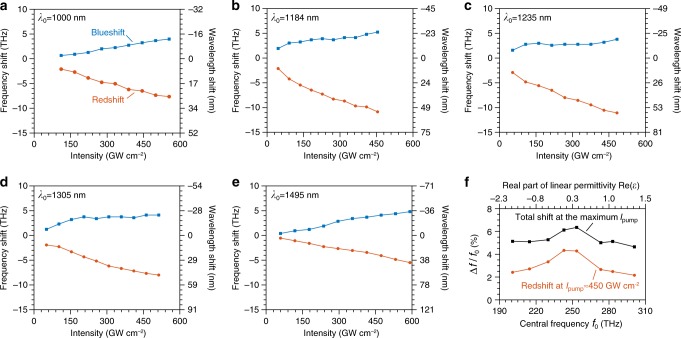
Wavelength-dependent time-refraction effect. **a**–**e** Experimentally measured maximum redshifts and blueshifts at different wavelengths *λ*_0_ as a function of peak pump intensities. **f** The red line denotes the fractional redshift |Δ*f*_red_|/*f*_0_ as a function of probe beam’s central frequency *f*_0_ at a peak pump intensity ~450 GW cm^−2^. The real part of the linear permittivity Re(*ε*) of ITO film at the corresponding central frequency *f*_0_ is shown in the top axis. The black line shows the total fractional shift (redshift plus blueshift) measured at the maximum pump intensities before the onset of higher-order nonlinear optical effects. We find that both the maximum fractional redshift and the total shift occur near the zero-permittivity wavelength.

Next, we investigate the dynamics away from the zero-permittivity wavelengths. We repeat the measurements at different excitation wavelengths from *λ*_0_ = 1000 nm−1500 nm. For each excitation wavelength and pump intensity, we extract the maximum frequency translation of the probe over a range of pump-probe delay time (see Supplementary Note 5). We summarize the wavelength- and intensity-dependent maximum frequency translations in Fig. 4a–e. Here, we limit the pump intensities to avoid the occurrence of significant higher-order nonlinearities. Our results reveal a number of trends. First, both the total achievable frequency translation (redshift and blueshift) and the maximum achievable redshift for a constant pump intensity are the highest near 1235 nm (where Re(*ε*) ≈ 0) than at other wavelengths. For example, at *λ*_0_ = 1495 nm the measured maximum magnitude of the redshift (5.4 THz) is a factor of two smaller than what can be achieved at *λ*_0_ = 1235 nm using a lower pump intensity. Nevertheless, we find that the total maximum fractional frequency translation (Δ*f*/*f*_0_) at near-zero permittivity is unprecedentedly large (Fig. 4f). The maximum total frequency translation of 14.9 THz (redshift of 11.1 THz and blueshift of 3.8 THz) at *λ*_0_ = 1235 nm (redshift plus blueshift) is over 53 times larger than what was achieved using a silicon ring resonator of a 6 μm diameter exhibiting a *Q*-factor greater than 18,000^20^. In contrast, the propagation distance in our material is only 620 nm which is 30 times shorter in physical length and four orders of magnitude smaller than the effective interaction length in a high-*Q* cavity. Moreover, our results show the operation bandwidth of ITO is much larger than what can be achieved using high-*Q* resonant structures.

## Discussion

As the refractive index of the ENZ material depends on the intensity of the pump, the work presented here may be formally described by cross-phase modulation with a delayed response^8^. However, the concept of time refraction is independent of the source type of the index change (e.g., thermally, optomechanically, or electrically induced index change) and is a more general effect than the simple cross-phase modulation that arises when the temporal boundary is specifically induced by an optical pulse. Furthermore, in contrast to a typical four-wave-mixing-based frequency conversion, the frequency shift obtained through time refraction does not depend on the frequency difference between the pump and the probe and is completely free from phase-mismatching. Although in this work the pump and the probe are frequency degenerate and produced from the same source using a beam splitter, it is not necessary for the beams to be frequency degenerate. Nevertheless, the maximum frequency shift with minimum energy expenditure can be achieved when both the pump and the probe lie within the ENZ spectral range. As the maximum index change happens at the zero-permittivity wavelength, the probe will undergo maximum frequency shift if its wavelength is at or near the zero-permittivity wavelength, whereas the energy expenditure will be minimum if the wavelength of pump is also at or near the zero-permittivity wavelength.

In conclusion, we have shown that a subwavelength-thick ITO film can be used to obtain unprecedentedly large (~6.5% of the carrier frequency), broadband and tunable frequency translation. The large time-refraction effect in the ENZ material raises the intriguing possibility of wavelength conversion over an octave using a time-varying ENZ medium. The magnitude of the frequency translation is primarily limited by the linear loss, higher-order nonlinear optical effects, dispersion, and the interplay between the pulse width and the interaction time. We note that the ENZ spectral region of ITO and other conducting oxides can be tuned at any wavelength between 1 µm and 3 µm by choosing the appropriate doping level^49,50^. Furthermore, because the effect is present in a bulk, homogeneous and isotropic material, one can engineer nanostructures incorporating ENZ media such as plasmonic waveguides, photonic crystal waveguides, and dynamic metasurfaces to arbitrarily control the sign and the magnitude of the frequency shift in order to build efficient octave-spanning frequency tuners while simultaneously lowering the required pump power by a few orders of magnitude^9^. For example, an appropriately engineered ITO-based platform can be used to shift an entire band of optical signals in the frequency domain. Such devices may find practical usage in quantum communication protocols requiring conversion of visible photons to infrared^51^ and in classical coherent optical communications^52,53^. We anticipate that the large time-refraction effect, we report here, can be exploited to engineer magnet-free nonreciprocal devices^54,55^, spatiotemporal metasurfaces^13^, and to investigate photonic time crystals and other topological effects in the time domain^16,56^ using free-space or on-chip ENZ-based structures.

## Methods

### Measurements

We use a tunable optical parametric amplifier (OPA) pumped by an amplified Ti:sapphire laser of ~120 fs for the experiments. The output of the OPA is split into two beams to produce the degenerate pump and probe beams using a pellicle beam splitter. Both beams are rendered *p*-polarized. The pump beam is focused onto the sample by a 25 cm lens yielding to a spot size of ~100 µm. The probe beam is focused by a 10 cm lens and its spot diameter is ~45 µm at 1235 nm. Although the spot size can change when the wavelength of the OPA output is adjusted, we always keep the probe beam spot size significantly smaller than the pump beam so that the probe beam experiences a nearly uniform change in the refractive index in the transverse dimensions. The angles of incidences are 15º and 10º for the pump and probe, respectively. The transmitted probe light is coupled to an optical spectrum analyzer via a multimode fiber with a 50 µm core diameter. The commercially available ITO thin film (PGO GmbH) has a thickness of 310 nm and is deposited on a 1.1-mm-thick glass substrate. We sandwich two such ITO films to make the 620 nm thick ITO sample by using a customized sample holder with adjustable tightening screws (See Supplementary Note 6). We use a translation stage to control the delay time between the pump and the probe beams. The experimental setup is presented in Supplementary Note 1.

## Supplementary information

Supplementary Information

Peer Review File

## Data Availability

All data supporting this study are available from the corresponding author upon request.

## Code availability

All relevant computer codes supporting this study are available from the corresponding author upon request.
