# Supplementary material for: Broadband frequency translation through time refraction in an epsilon-near-zero material
Source: Nat Commun. 2020 May 1;11:2180. doi: 10.1038/s41467-020-15682-2 (PMC7195366; doi:10.1038/s41467-020-15682-2)
Supplement: Supplementary file 1 — Supplementary Information [file 41467_2020_15682_MOESM1_ESM.pdf]

Supplementary Information

Broadband frequency translation through time refraction in an epsilon-near-zero material

Zhou *et al*.

Supplementary Information

Yiyu Zhou[1,†,*], M. Zahirul Alam[2, †], Mohammad Karimi[2], Jeremy Upham[2], Orad Reshef[2], Cong Liu[3], Alan E. Willner[3] and Robert W. Boyd[1,2]

[1]The Institute of Optics, University of Rochester, Rochester, New York 14627, USA

[2]Department of Physics, University of Ottawa, Ottawa, Ontario K1N 6N5, Canada

[3]Department of Electrical Engineering, University of Southern California, Los Angeles, California, 90089, USA

[†]These authors contributed equally

[*]corresponding author: yzhou62@ur.rochester.edu

Supplementary Note 1: Experimental setup

We use a wavelength-tunable optical parametric amplifier (TOPAS prime, Spectra Physics) pumped by an amplified Ti:sapphire laser (Mai Tai, Spectra Physics) as the ultrafast pulse source. A 45:55 pellicle beamsplitter (BP245B3, Thorlabs) is used to split the input beam, and a high-precision translation stage (DDSM100, Thorlabs) is used to tune the delay time between pump and probe pulses. We use a thin film polarizer mounted on a rotation stage and a Glan-Taylor polarizing beam splitter to control the pulse intensity while keeping the polarization to be p-polarized. We use a multimode fiber with 50 μm core diameter to collect the probe pulse after the sample and use an optical spectrum analyzer to record the spectra of the probe.

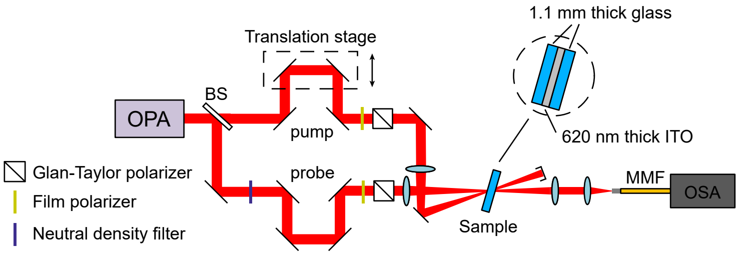

Supplementary Figure 1. The schematic of the experimental setup. In the pump beam arm, we rotate the first polarizer to control the peak pump intensity. BS, pellicle beamsplitter. OPA, optical parametric amplifier. OSA, optical spectrum analyzer. MMF, multimode fiber.

Supplementary Note 2: Numerical simulation

We use a split-step Fourier method to solve the nonlinear Schrödinger equation (NLSE)[1] to model the nonlinear interactions between the pump and the probe beam pulses propagating through the ITO sample. We ignore all nonlinear optical effects higher than the third-order effects[2]. The propagation of the probe pulse through the time-varying ITO can be described as[3]

$$\frac{\partial A}{\partial z} + \beta_1 \frac{\partial A}{\partial t} + \mathrm{i}\frac{\beta_2}{2}\frac{\partial^2 A}{\partial t^2} = \mathrm{i}\frac{\omega_0}{c}\Delta n_{\mathrm{eff}}(t - t_{\mathrm{d}}, I_{\mathrm{pump}}(t))A, \quad (1)$$

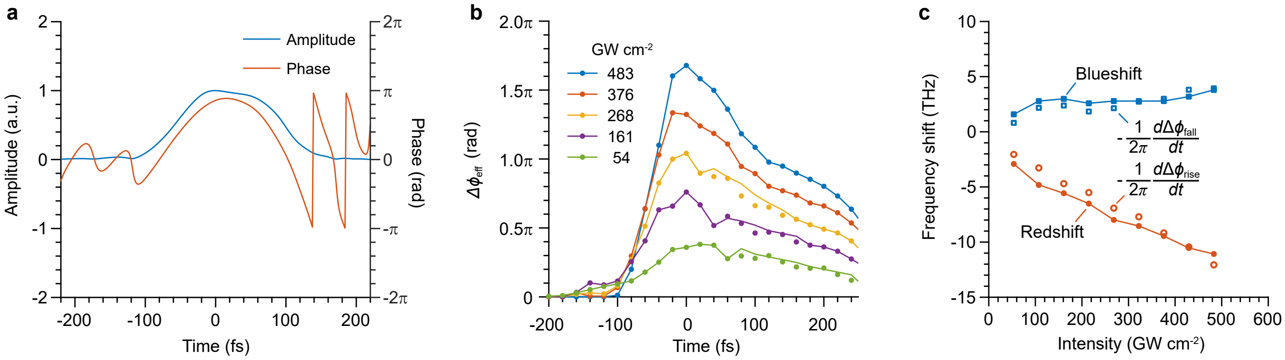

Supplementary Figure 2. **a**, Amplitude and phase of the optical pulses at 1235 nm retrieved by frequency-resolved optical gating. **b**, Numerically retrieved nonlinear phase variations $\Delta\phi_{\mathrm{eff}}(t)$ at different pump intensities. The dots represent the numerical results and the solid lines connecting the dots are to facilitate visualization. **c**, The experimentally measured redshifts and blueshifts as a function of pump intensity are represented by the solid lines, and the calculated maximum local frequency shifts are represented by hollow circles.

where $A(z,t)$ is the slowly varying envelope of the probe pulse, $\beta_1 \equiv (1/v_{\mathrm{g}})$ is the inverse of the group velocity, $\beta_2 \equiv \frac{\partial}{\partial\omega}(1/v_{\mathrm{g}})$ is the group velocity dispersion, c is the speed of light in vacuum, t_{d} is the pump-probe delay time, and ω_0 is the angular frequency of the light beams. The effective time-dependent refractive index index $\Delta n_{\mathrm{eff}}(t, I_{\mathrm{pump}})$ on the right-hand side is a function of pump intensity and the delay time between the pump and the probe. This term acts as the driving term for the nonlinear pulse propagation and is responsible for the frequency translation. The relative permittivity of ITO around its epsilon-near-zero spectral range can be modeled by a Drude function as[4]

$$\varepsilon(\omega) = \varepsilon_\infty - \omega_{\mathrm{p}}^2/(\omega^2 + \mathrm{i}\gamma\omega), \tag{2}$$

where $\varepsilon_\infty = 3.80$, $\omega = 2\pi c/\lambda$ is the frequency of light, $\omega_{\mathrm{p}} = 2\pi \cdot 473$ THz is the plasma frequency, and $\gamma = 0.0468 \cdot 2\pi \cdot 473$ THz is the damping rate. The refractive index of ITO can thus be expressed as $n(\omega) = \sqrt{\varepsilon(\omega)}$. The dispersion coefficients can be calculated as $\beta_m = \left(\frac{d^m\beta}{d\omega^m}\right)_{\omega=\omega_0}$, where $\beta(\omega) = \mathrm{Re}(n(\omega))\omega/c$. At $\lambda = 1235$ nm we have $\beta_1 = 1.53 \times 10^7$ fs m^{-1} and $\beta_2 = 1.10 \times 10^8$ fs^2 m^{-1}. The dispersion length is found to be $L_{\mathrm{D}} = T_0^2/\beta_2 = 47.1$ μm, where $2\sqrt{\ln(2)}T_0 = 120$ fs is the FWHM pulse width. Since the dispersion length is two orders of magnitude larger than the ITO thickness of 0.62 μm, we neglect higher-order dispersion terms.

For the numerical simulation we ignore the changes in $\Delta n_{\mathrm{eff}}(t)$ that occur at the time scale of the oscillation of the carrier waves of the pump pulse. We only take into account the pump-envelope-dependent changes in the refractive index. Thus, in our simulation we approximate $\Delta n_{\mathrm{eff}}(t)$ as a function of the envelope of the pump pulse only. We use an iterative least squares curve fitting algorithm to extract an approximate shape of pump-intensity-dependent $\Delta n_{\mathrm{eff}}(t)$ that results in the experimentally obtained output probe spectra for a fixed pump intensity as follows: (1) We begin with an initial guess $\Delta n_{\mathrm{eff}}^1(t)$. For j-th iteration, the corresponding index change is denoted as $\Delta n_{\mathrm{eff}}^j(t)$. (2) For the index change $\Delta n_{\mathrm{eff}}^j(t)$ and a specific pump-probe delay time t_{d}', we use $\Delta n_{\mathrm{eff}}^j(t - t_{\mathrm{d}}')$ in Supplementary Eq. (1) and perform the split-step Fourier method to generate an output pulse $A^j(z_1, t; t_{\mathrm{d}}')$,

where $z_1 = 620$ nm. The output spectrum can be calculated via Fourier transform as $S^j(f, t'_d) = |\int A^j(z_1, t; t'_d) \exp(-i2\pi ft)\, dt|^2$. We repeat this procedure for different delay times and therefore obtain the two-dimensional spectrogram $S^j(f, t_d)$ with -200 fs $\le t_d \le 200$ fs. (3) We use a fitting algorithm (lsqcurvefit, MATLAB) to fit $S^j(f, t_d)$ to the experimental data $S^{exp}(f, t_d)$ by adjusting $\Delta n^j_{eff}(t)$. In other words, $\Delta n^j_{eff}(t)$ is the parameter to be tuned iteratively. The final results of $S^j(f, t_d)$ at different pump intensities are presented in Fig. 3(d-f) in the manuscript.

In order to obtain the temporal amplitude and phase of the input probe pulses at the wavelengths of interest, i.e. $A(z = 0, t)$, we performed a series of frequency-resolved optical gating (FROG) measurements[5]. In our simulation we always assume a homogeneous index change throughout the entire ITO film for simplification. We also ignore the time difference of the index change between the front and the back ends of the sample since the transit time through the sample is roughly two-orders of magnitude smaller than the temporal width of the pump pulse. We also ignore the Fresnel-reflection- and absorption-induced change in the pump intensity inside the sample for simplification. In addition, we also ignore nonlinear change in dispersion. The experimentally measured temporal amplitude and phase of the input probe pulse at 1235 nm is shown in Supplementary Fig. 2a.

An alternative way to understand the effect of index change is to use the concept of a local frequency shift. The local frequency shift can be used as an approximate estimation of spectral shift and can be expressed as[6]

$$\Delta f(t) = \frac{1}{2\pi}\Delta\omega(t) = -\frac{1}{2\pi}\frac{d\Delta\phi_{eff}(t)}{dt}, \tag{3}$$

where the nonlinear phase variation induced by Δn_{eff} is $\Delta\phi_{eff}(t) = k \cdot L \cdot \Delta n_{eff}(t)$, where $k = 2\pi/\lambda$ is the wavenumber, and $L = 620$ nm is the ITO thickness. The retrieved phase variation $\Delta\phi_{eff}$ is displayed for different peak pump intensities in Supplementary Fig. 2b. For a given $\Delta\phi_{eff}$, the maximum local frequency redshift is determined by the rising edge of the nonlinear phase change as $\Delta f_{red} = \min(\Delta f(t)) = -\frac{1}{2\pi}\frac{d\Delta\phi_{rise}}{dt}$, while the maximum local frequency blueshift is determined by the falling edge as $\Delta f_{blue} = \max(\Delta f(t)) = -\frac{1}{2\pi}\frac{d\Delta\phi_{fall}}{dt}$. The numerically retrieved maximum local frequency shifts at different pump intensities are shown in Supplementary Fig. 2c. The retrieved maximum local frequency shifts are in good agreement with the experimentally measured frequency shifts.

Supplementary Note 3: Additional time refraction effects and unnormalized spectra

Since the focus of this work is on frequency translation, we presented spectral data with normalized amplitude in the main text. However, time refraction also affects the amplitude of the frequency-translated pulse[7]. Let us consider the simplest time-refraction model — a Gaussian pulse with carrier frequency ω_0 and pulse width T_0 is travelling through a medium whose

refractive index changes from n_1 to n_2. We assume that the longitudinal length of the medium is larger than the longitudinal length of the pulse. In such an idealized case the input (E_{in}) and output (E_{out}) pulses can be expressed as[8]

$$E_{\mathrm{in}} = E_0 \exp(-\frac{t^2}{2T_0^2}) \exp(-\mathrm{i}\omega_0 t), \qquad E_{\mathrm{out}} = (\frac{n_1}{n_2} E_0)\ \exp(-\frac{t^2}{2(T_0 n_2/n_1)^2})\ \exp(-\mathrm{i}\frac{n_1}{n_2}\omega_0 t). \tag{4}$$

The above simple model shows that the time refraction effect can modify amplitude, temporal width (by Fourier relation the spectral width), and the central frequency. Therefore, the relative changes in spectral amplitude may also be of interest. Below we present the spectral data for five different probe wavelengths for various pump-probe delays in Supplementary Fig. 3-7, where the spectral peak magnitude in the absence of pump pulse is normalized to unity. However, it should be noted that in addition to time refraction there are several other competing effects in ITO that can significantly modulate the transmittance of the probe pulse. Since the ITO has a small linear index and exhibits a large nonlinear index change, the Fresnel reflection coefficients exhibit strong time-dependent variations. In addition, due to saturable absorption, the imaginary part of the refractive index and, consequently, the loss in ITO can drop significantly in the presence of a pump pulse. Thus the spectral amplitude of the probe for a given pump-probe delay and pump intensity depends on time-refraction effects, nonlinear changes in Fresnel reflection coefficient, and changes in absorption. Therefore a sophisticated model is required to isolate these three effects to accurately quantify the effect of time refraction on the amplitude, which is beyond the scope of this work. Nevertheless, it can be noticed that for all wavelengths of interest, the transmittance of the probe beam significantly increases when the probe is blueshifted.

We also note that the absorption loss in ITO in the ENZ spectral range is large compared to standard dielectric materials. We note that a fair figure of merit for such a medium should be the magnitude of nonlinear change over one absorption length. Clearly ITO cannot be used to make optical fibre or nanophotonics waveguides of hundreds of microns in length. The crucial advantage of these ENZ materials over other conventional materials is that one needs to propagate over only a sub-micron distance in a nonlinear ENZ material for a comparable effect that is achieved by a hundreds of microns long nanophotonic waveguide made of a conventional photonics material. It should be noted that for this work we are using a commercially available sample that has never been optimized for the purpose of this work. In addition, there are still many opportunities to further enhance the performance of ENZ materials. A few promising routes are: i) use an ENZ material with higher electron mobility, ii) nanostructure the ENZ material or include plasmonic systems to achieve higher effective index change within reduced thickness and loss, iii) integrate ENZ material with optical waveguides as the cladding, etc. We also note that in the presence of strong pump the transmittance of the probe can actually increase due to the increased refractive index and reduced loss. Finally, we note that the use of optical amplifiers to boost a weak signal is a standard system protocol. Furthermore, the recent advancement in short pulse amplification might also be of interest for particular applications[9].

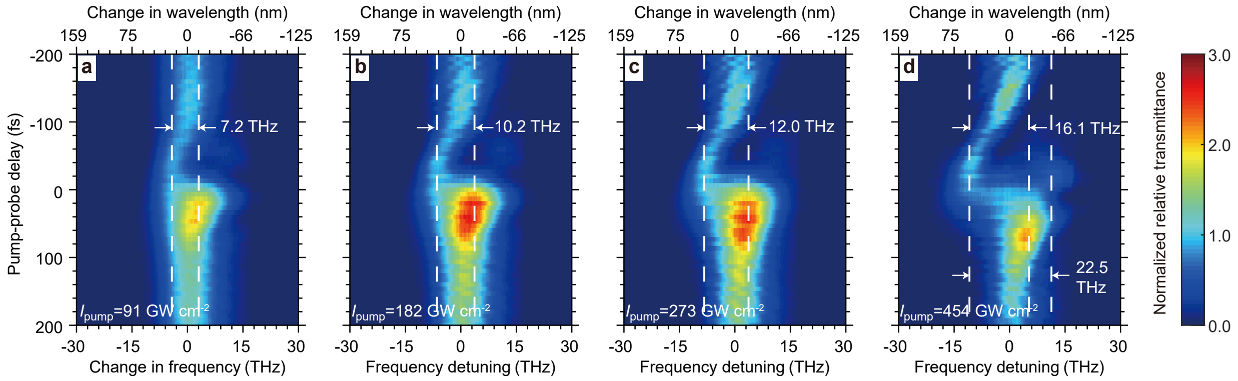

Supplementary Figure 3. **a-d**, The experimentally measured spectra of 1184 nm probe pulse at different pump intensities. The spectral peak of the probe beam in the absence of pump beam is normalized to unity.

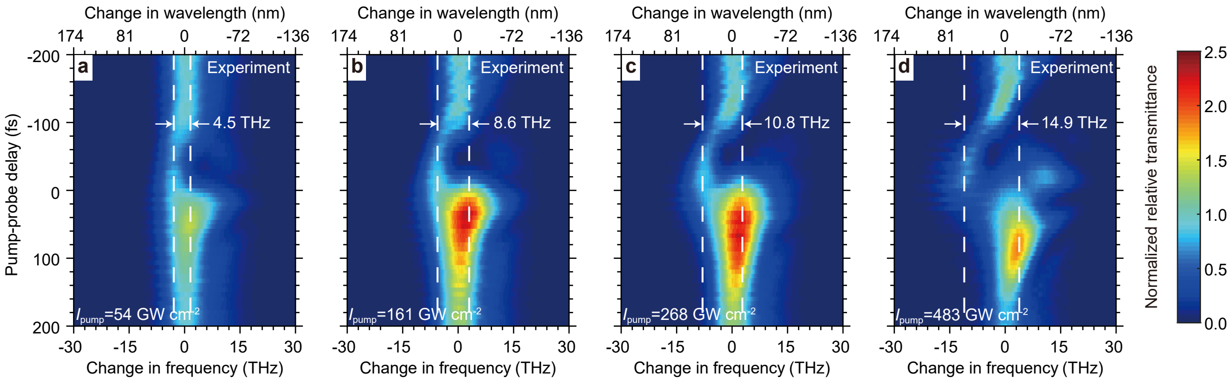

Supplementary Figure 4. **a-d**, The experimentally measured spectra of 1235 nm probe pulse at different pump intensities. The spectral peak of the probe beam in the absence of pump beam is normalized to unity.

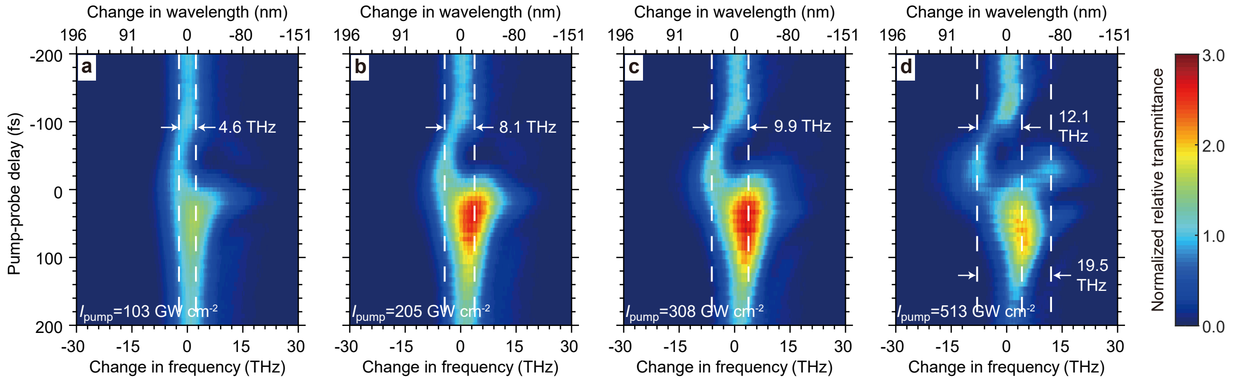

Supplementary Figure 5. **a-d**, The experimentally measured spectra of 1305 nm probe pulse at different pump intensities. The spectral peak of the probe beam in the absence of pump beam is normalized to unity.

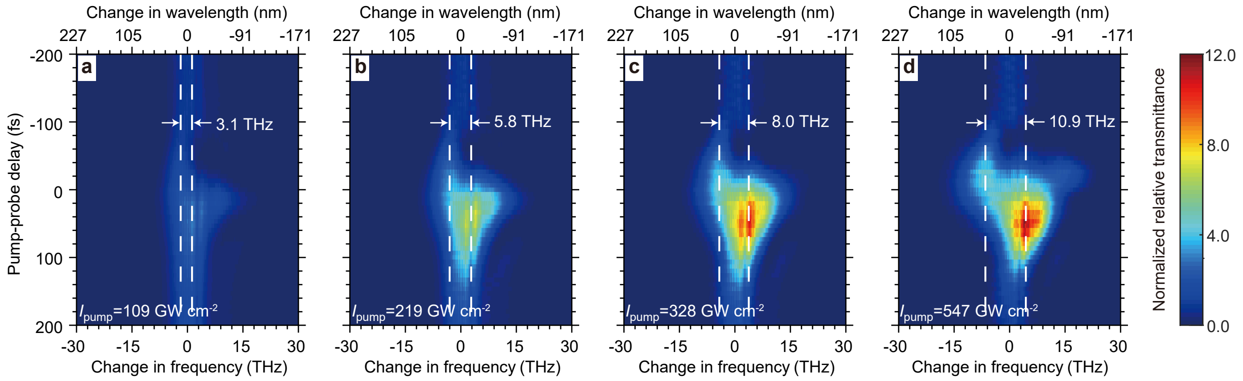

Supplementary Figure 6. **a-d**, The experimentally measured spectra of 1398 nm probe pulse at different pump intensities. The spectral peak of the probe beam in the absence of pump beam is normalized to unity.

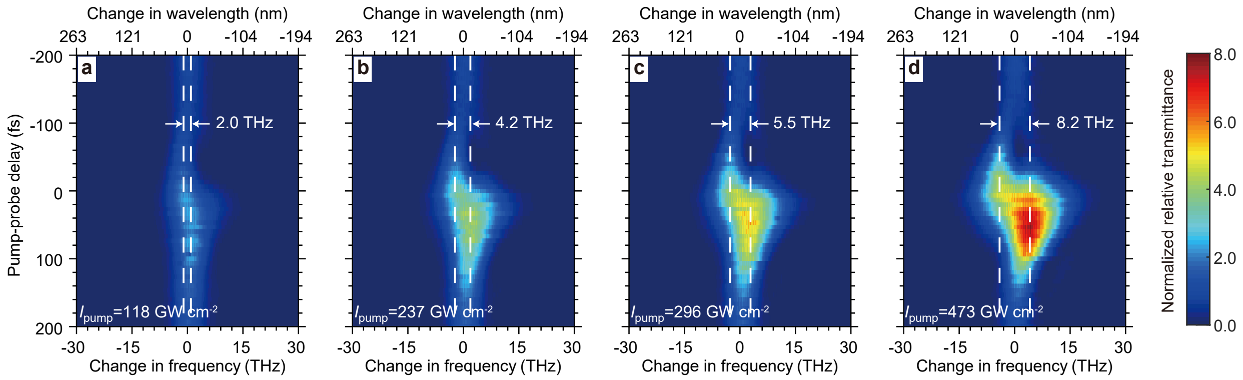

Supplementary Figure 7. **a-d**, The experimentally measured spectra of 1495 nm probe pulse at different pump intensities. The spectral peak of the probe beam in the absence of pump beam is normalized to unity.

Supplementary Note 4: Effects of fifth-order nonlinearity

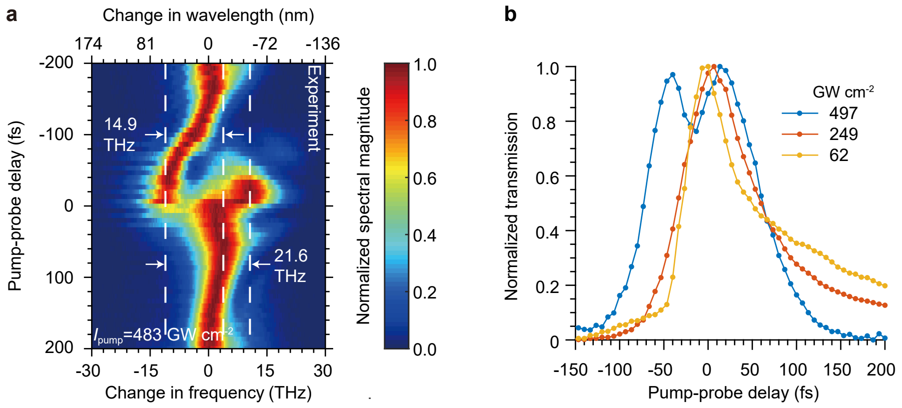

Supplementary Figure 8. **a**, The experimentally measured probe spectrogram at the peak pump intensity of 483 GW cm^{-2}. **b**, The experimentally measured probe transmission at different pump intensities as a function of pump-probe delay time.

At a high pump intensity of greater than 450 GW cm^{-2} close to the zero permittivity wavelength we observe onsets of higher-order nonlinear optical effects. For example, at $\lambda_0 = 1235$ nm at a pump intensity of 480 GW cm^{-2} we observe a relatively large blueshifted peak as shown in Supplementary Fig. 8a due to an effective fifth-order nonlinear optical process. In a pump-probe transmission measurement this effective fifth-order process leads to a sudden drop in the transmission of the probe Supplementary Fig. 8b. From these results we conclude that the effective fifth-order process has an opposite sign to that of the effective third-order process and that the relevant time scales for the fifth-order-nonlinearity-induced blue shift is much shorter compared to the effective third-order-nonlinearity-induced blue shift at lower intensities.

Supplementary Note 5: Normalized spectra at other wavelengths

Here, we provide the detailed measurement results for pump-probe wavelengths ranging from $\lambda = 1000$ nm to 1500 nm in Supplementary Fig. 9-14.

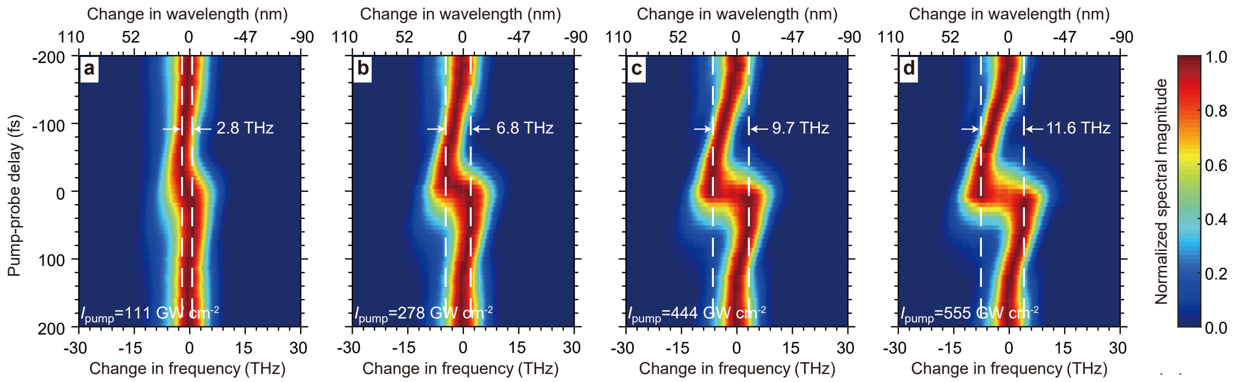

Supplementary Figure 9. **a-d**, The experimentally measured spectra of 1000 nm probe pulse at different pump intensities. The magnitudes of the measured maximum redshift and blueshift are denoted by the corresponding white dashed lines.

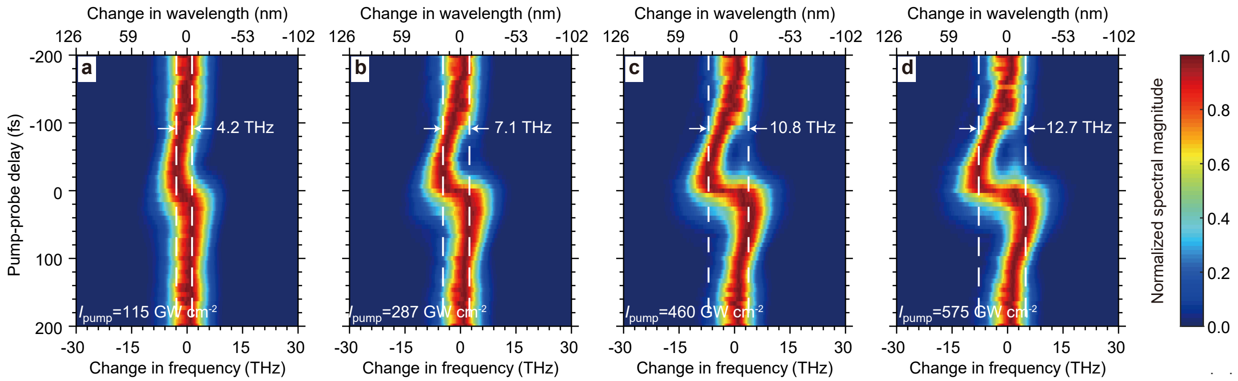

Supplementary Figure 10. **a-d**, The experimentally measured spectra of 1060 nm probe pulse at different pump intensities. The magnitudes of the measured maximum redshift and blueshift are denoted by the corresponding white dashed lines.

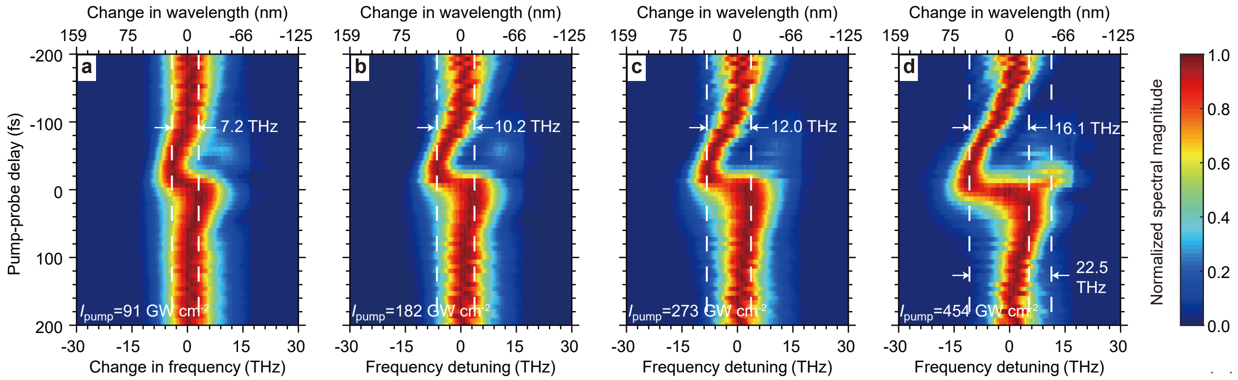

Supplementary Figure 11. **a-d**, The experimentally measured spectra of 1184 nm probe pulse at different pump intensities. The magnitudes of the measured maximum redshift and blueshift are denoted by the corresponding white dashed lines. The subpanel **d** also shows the an additional blueshifted peak (6.4 THz) due to the effective fifth-order nonlinear process.

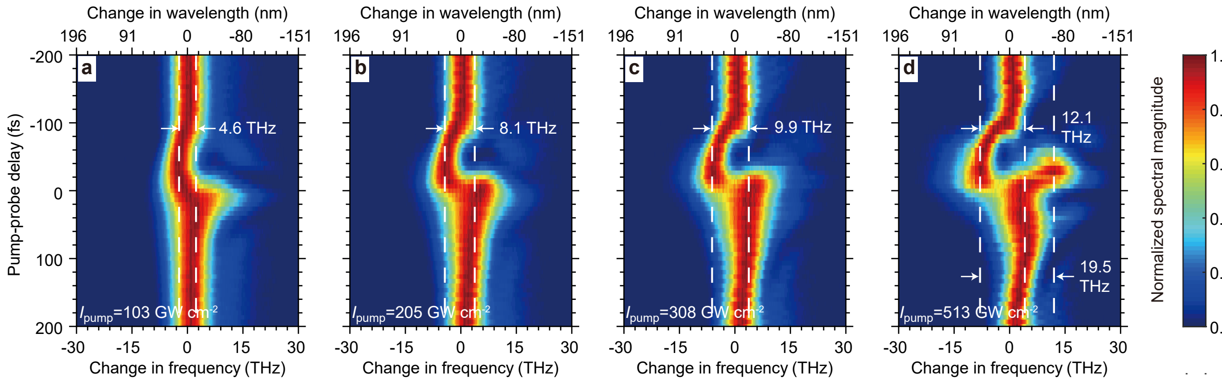

Supplementary Figure 12. **a-d**, The experimentally measured spectra of 1305 nm probe pulse at different pump intensities. The magnitudes of the measured maximum redshift and blueshift are denoted by the corresponding white dashed lines.The subpanel **d** also shows the an additional blueshifted peak (7.4 THz) due to the effective fifth-order nonlinear process.

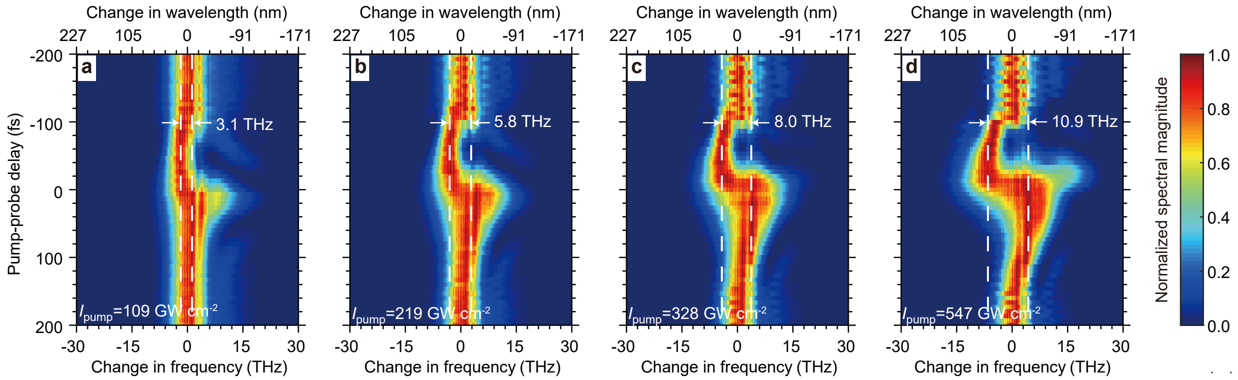

Supplementary Figure 13. **a-d**, The experimentally measured spectra of 1398 nm probe pulse at different pump intensities. The magnitudes of the measured maximum redshift and blueshift are denoted by the corresponding white dashed lines.

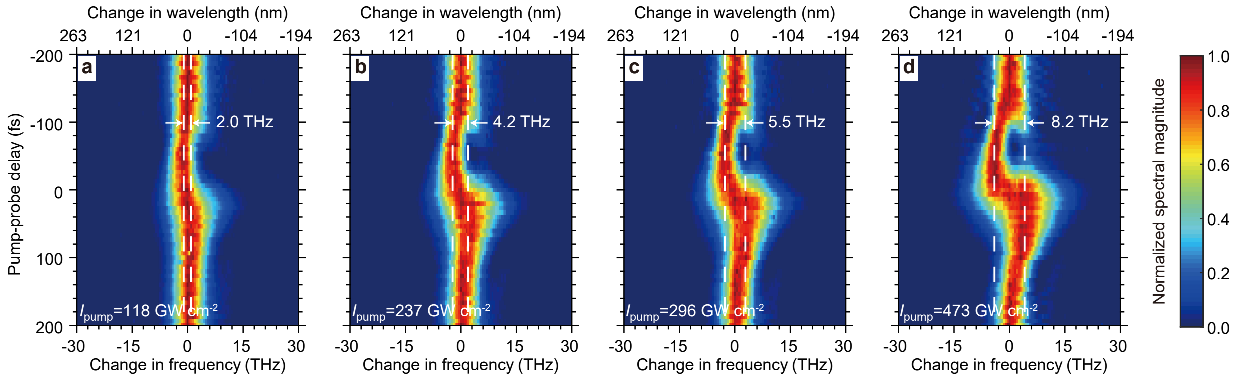

Supplementary Figure 14. **a-d**, The experimentally measured spectra of 1495 nm probe pulse at different pump intensities. The magnitudes of the measured maximum redshift and blueshift are denoted by the corresponding white dashed lines.

Supplementary Note 6: Analysis of the air gap between ITO films

In our experiment, we form a 620-nm-thick medium by sandwiching two commercially available 310-nm-thick ITO films deposited on 1.1-mm-thick glass. However, a white-light interferometric measurement reveals that there remains an air gap between two layers of the ITO thin films. We measure the air gap to be roughly 2200 nm thick. Air has a higher refractive index than ITO at the wavelengths of interest (e.g. $n_{\mathrm{ITO}} = 0.42 + 0.42\mathrm{i}$ at $\lambda_0 = 1240$ nm) and the air gap has a thickness slightly larger than the free-space wavelength. Thus one may expect that there is an unintended weak cavity effect due to thin film interference effects for our structure. However we posit that this unintended air gap affects our results minimally for two reasons. First, compared to ITO the nonlinear response of the air gap at the pump intensities relevant for this experiment is negligible. Second, the effective third-order nonlinear response of ITO leads to an increase in refractive index at all wavelengths of interest in this work. Thus at high pump intensities for which the frequency translation of the probe pulses are the largest, the thin-film interference effects effectively disappears.

Supplementary References

1. Agrawal, G. P. *Nonlinear fiber optics* (Springer, 2000).

2. Boyd, R. W. *Nonlinear optics* (Elsevier, 2003).

3. Plansinis, B. W., Donaldson, W. R. & Agrawal, G. P. What is the temporal analog of reflection and refraction of optical beams? *Phys. Rev. Lett.* **115**, 183901 (2015).

4. Alam, M. Z., De Leon, I. & Boyd, R. W. Large optical nonlinearity of indium tin oxide in its epsilon-near-zero region. *Science* **352**, 795–797 (2016).

5. Trebino, R. et al. Measuring ultrashort laser pulses in the time-frequency domain using frequency-resolved optical gating. *Rev. Sci. Instrum.* **68**, 3277–3295 (1997).

6. Dekker, R. et al. Ultrafast kerr-induced all-optical wavelength conversion in silicon waveguides using 1.55 μm femtosecond pulses. *Opt. Express* **14**, 8336–8346 (2006).

7. Xiao, Y., Maywar, D. N. & Agrawal, G. P. Reflection and transmission of electromagnetic waves at a temporal boundary. *Opt. Lett.* **39**, 574–577 (2014).

8. Xiao, Y. *Propagation of optical pulses in dynamic media: a time transformation method* (PhD thesis, University of Rochester, 2014).

9. Vampa, G. et al. Light amplification by seeded kerr instability. *Science* **359**, 673–675 (2018).
