# Supplementary material for: Broadband frequency translation through time refraction in an epsilon-near-zero material
Source: Nat Commun. 2020 May 1;11:2180. doi: 10.1038/s41467-020-15682-2 (PMC7195366; doi:10.1038/s41467-020-15682-2)
Supplement: Supplementary file 2 — Peer Review File [file 41467_2020_15682_MOESM2_ESM.pdf]

Editorial Note: This manuscript has been previously reviewed at another journal that is not operating a transparent peer review scheme. This document only contains reviewer comments and rebuttal letters for versions considered at Nature Communications.
